# Pediatric doxorubicin exposure induces persistent pathological changes in mice

**DOI:** 10.1016/j.taap.2025.117600

**Published:** 2025-10-14

**Authors:** Jingrui Chen, Mahder Dawit Belew, Jing Wei, Eric J. Chow, Zhaokang Cheng

**Affiliations:** a Department of Pharmaceutical Sciences, Washington State University, 412 E. Spokane Falls Blvd., Spokane, WA 99202-2131, USA; b Clinical Research and Public Health Sciences Divisions, Fred Hutchinson Cancer Center, PO Box 19024, Mailstop M4-C308, Seattle, WA 98109, USA

**Keywords:** Cancer Survivorship, Growth Delay, Cardiomyopathy, Heart Mass, Hepatotoxicity

## Abstract

Over 50 % of pediatric cancer patients undergo treatment with chemotherapy regimens containing anthracyclines, such as doxorubicin (DOX). However, the long-term effects of childhood DOX exposure remain poorly understood, and protective strategies are limited. To establish a mouse model that recapitulates the chronic health conditions in adult survivors of childhood cancer, 14-day-old C57BL/6N mice received DOX (2.5 mg/kg, twice weekly for 2 weeks, i.p.) and were monitored for 32 weeks. Pediatric DOX injection induced late cardiotoxicity including systolic and diastolic dysfunction, cardiac fibrosis and cardiomyocyte atrophy, which were alleviated by treatment with the CDK7/12/13 inhibitor THZ1. Pediatric DOX also reduced heart, liver and spleen weight, while sparing the lung and kidney. Mechanistically, DOX induced persistent activation of p38 in the heart and diminished physiological cardiomyocyte hypertrophy. Pediatric DOX caused slow body weight gain and late mortality, which were surprisingly exacerbated by THZ1. Notably, pediatric THZ1 exposure also hindered body weight gain and reduced heart and liver weight. In conclusion, pediatric DOX exposure resulted in chronic cardiac dysfunction, underweight and premature death during adulthood in mice. Pharmacologic inhibition of CDK7/12/13 with THZ1 partially protected against pediatric DOX-induced cardiotoxicity, but aggravated growth delay and accelerated mortality.

## 1. Introduction

In the United States, nearly ten thousand children under the age of 15 are diagnosed with cancer each year (Siegel et al., 2024). Because of the advances in therapies, over 85 % of children are expected to survive at least five years past their initial cancer diagnosis (Siegel et al., 2024). Unfortunately, childhood cancer survivors often suffer from chronic health conditions later in life. These cancer survivors have a ~ 10-fold higher risk of dying from cardiovascular diseases, the most common cause of noncancer mortality in this population (Lipshultz et al., 2013). For childhood cancer survivors, cardiovascular diseases are often caused by cardiotoxic cancer therapies such as anthracyclines (Lipshultz et al., 2013). According to the Childhood Cancer Survivor Study, over 60 % of long-term survivors have been exposed to chemotherapy containing anthracyclines (Mulrooney et al., 2020). Depending on the time between therapy and the onset of symptoms, anthracyclines are known to cause acute cardiotoxicity (occurring within one week of therapy), early-onset (within one year) or late-onset chronic cardiotoxicity (after one year) (Lipshultz et al., 2013). Late-onset cardiotoxicity can occur decades after anthracycline exposure, as a result of long-term cardiac remodeling following the initial myocardial injury. With the rapid increase in cancer survivors who are still alive >20years after diagnosis, late effects of cancer therapies are expected to be observed more frequently in the clinic and pose a major challenge to the health-care system (Tonorezos et al., 2024a).

Over the past decades, cardiovascular toxicity caused by anthracyclines such as doxorubicin (DOX) has attracted significant attention. Preclinical studies have revealed multiple cardiotoxic mechanisms and identified potential cardioprotective strategies. It has been increasingly recognized that children have a developing cardiovascular system that is particularly vulnerable to cancer therapies (Lipshultz et al., 2013). Still, there is a paucity of preclinical research that accurately mimics the chronic cardiotoxicity in adult survivors of childhood cancer (Armenian et al., 2018). We recently reported that THZ1, a covalent inhibitor for cyclin-dependent kinase (CDK) 7/12/13, attenuated DOX-induced cardiomyopathy in adult mice (Chen et al., 2024). In the present study, we developed a clinically relevant mouse model of pediatric DOX-induced late cardiotoxicity and investigated the protective effect of CDK7/12/13 inhibition.

## 2. Methods

### 2.1. Animals

For in vivo studies, the juvenile mice were generated by breeding male and female C57BL/6N mice (Inotiv, Inc). For in vitro studies, the neonatal rats were generated by breeding male and female Sprague-Dawley rats (Inotiv, Inc). Anesthesia was achieved by inhalation of isoflurane (1–5 %). Animals were euthanized by CO2 inhalation followed by a secondary physical method including cervical dislocation or decapitation. All animal studies were approved by the Institutional Animal Care and Use Committee at Washington State University (#6480).

### 2.2. Pediatric mouse model of long-term DOX cardiotoxicity

**Fig. 1.**
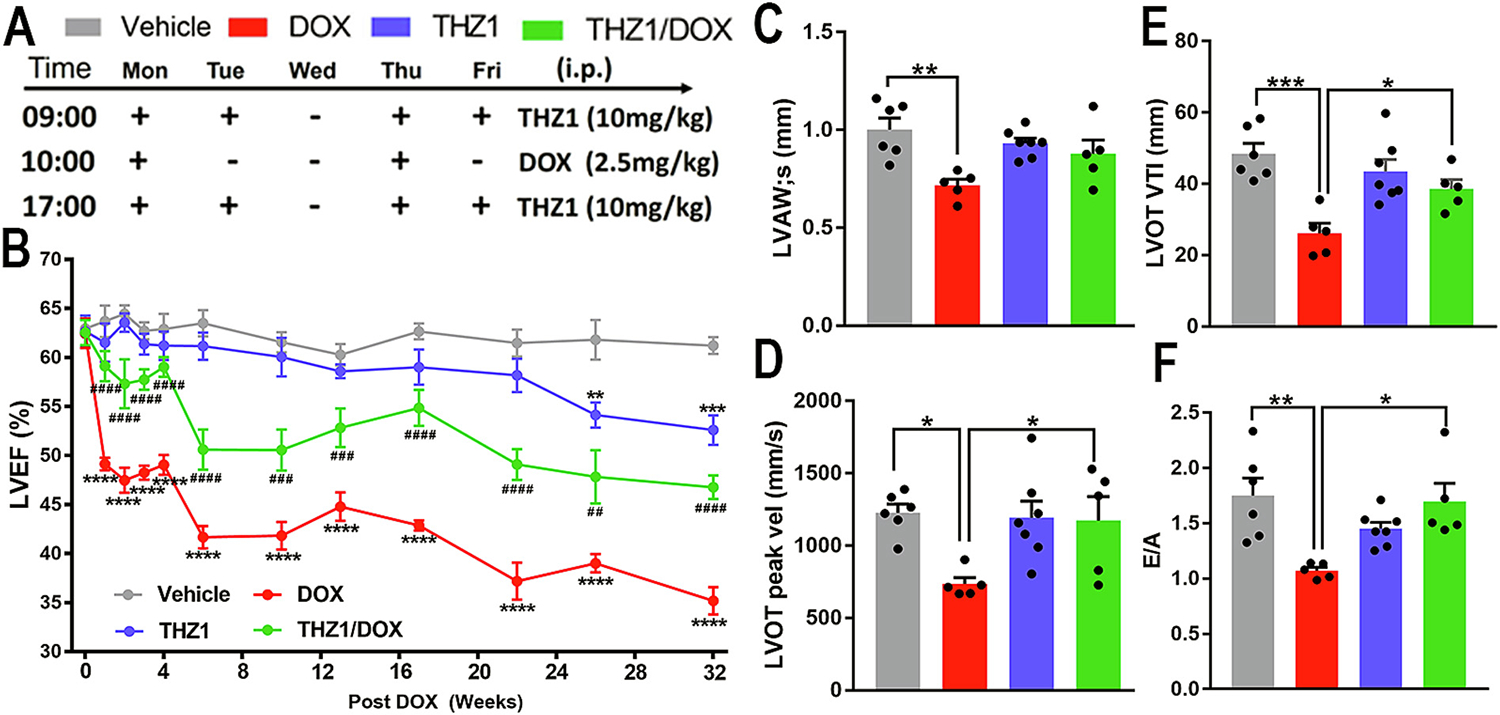
Pediatric DOX exposure-induced long-term cardiac dysfunction was attenuated by THZ1. (A) Experimental protocol. C57BL/6N mice (age 14 days, male) received injections of DOX (2.5 mg/kg, i.p., twice weekly) and/or the CDK7/12/13 inhibitor THZ1 (10 mg/kg, i.p. twice daily, 4 days per week) for 2 weeks. Vehicle (*n* = 6), DOX (*n* = 7), THZ1 (n = 7), THZ1/DOX (n = 7). (B-F) Heart function was monitored using echocardiography for 32 weeks. (B) LV ejection fraction (LVEF). Vehicle (n = 6), DOX (*n* = 5–7), THZ1 (n = 7), THZ1/DOX (n = 5–7). Data were analyzed using two-way ANOVA with Tukey’s test. ** *P* < 0.01, *** *P* < 0.001, **** *P* < 0.0001 vs Vehicle. ## *P* < 0.01, ### *P* < 0.001, #### *P* < 0.0001 vs DOX. (C) LV end-systolic anterior wall thickness (LVAW; s) at week 32. (D) LV outflow tract peak velocity (LVOT peak vel) at week 32. (E) LV outflow tract velocity time integral (LVOT VTI) at week 32. (F) Ratio of E-wave to A-wave (E/A) at week 32. Vehicle (n = 6), DOX (n = 5), THZ1 (n = 7), THZ1/DOX (n = 5). Two-way ANOVA with Tukey’s test. * *P* < 0.05, ** *P* < 0.01, *** *P* < 0.001.

Fourteen-day-old male C57BL/6N mice received injections of DOX (2.5 mg/kg, twice weekly for 2 weeks, i.p., cumulative dose 10 mg/kg) and/or the CDK7/12/13 inhibitor THZ1 (10 mg/kg, twice daily, 4 days per week for 2 weeks, i.p., Fig. 1A). For each DOX injection, THZ1 was administered 1 h before and 7 h after DOX within the same day, then twice again at the same times on the following day. Mice were randomly divided into four groups: 1) vehicle (10 % DMSO in D5W and saline); 2) DOX; 3) THZ1; or 4) THZ1 / DOX. To prevent local tissue damage, a different area of the peritoneal cavity was targeted for injection at each time point. No overt inflammation or damage was apparent at the injection sites. All mice were euthanized at 32 weeks (~8 months) after the first DOX injection (i.e. 34 weeks of age).

### 2.3. Echocardiography

Heart function was monitored using echocardiography under anesthesia with isoflurane. Briefly, two-dimensional short-axis views were obtained in M mode using the Vevo 2100 imaging system (VisualSonics) while the heart rate is between 400 and 500 bpm. The left ventricular (LV) outflow tract flow and mitral flow are assessed in pulsed-wave (PW) Doppler mode.

### 2.4. Histology

At week 32 post DOX injection, major organs including heart, liver, spleen, lung and kidney were harvested. The wet weight of each organ and the tibia length were measured. The heart was rinsed with PBS and then fixed in 4 % PFA for 24 h, followed by dehydration, paraffin embedding and sectioning. Myocardial fibrosis was evaluated by Masson’s trichrome staining (HT10516, Sigma). Cardiomyocyte cross-sectional area was evaluated by wheat germ agglutinin staining (W32464, Invitrogen). Cardiomyocyte size was measured from at least 50 cells per heart using ImageJ.

### 2.5. Cell culture

Neonatal rat cardiomyocytes (NRCMs) were isolated from 2- to 4-day-old Sprague-Dawley rats as described previously (Chen et al., 2024). NRCMs were cultured in serum-free Medium 199 supplemented with 0.5 % penicillin/streptomycin. NRCMs were incubated with DOX (0.5 μM), which is comparable to the peak plasma concentration of 0.3–6 μg/ml (0.5 – 10 μM) after a single dose of DOX (25 – 75 mg/m^2^) in cancer patients (Bramwell et al., 2002; Hempel et al., 2002; Wilde et al., 2007). Physiological hypertrophy was induced by treatment with insulin-like growth factor 1 (IGF1, 100 ng/ml, Proteintech) for 48 h. Cells were then subjected to immunofluorescent staining with mouse anti-cardiac Troponin T antibody (1:100, MS-295-P, Thermo Scientific) and DAPI (300 nM, D1306, Thermo Fisher).

### 2.6. Western blotting

Western blotting was performed using the following primary antibodies: phospho-p38 (Thr180/Tyr182, 1:1000, #4511, Cell Signaling Technology), p38 (1:1000, #9212, Cell Signaling Technology), Lamin B (1:1000, 12987–1-AP, Proteintech), and α-Tubulin (1:1000, T6074, Sigma-Aldrich).

### 2.7. Statistical analysis

Results were expressed as mean ± SEM and analyzed using Graph-Pad Prism 7. Statistical comparisons were performed using Student’s *t*-test, one-way analysis of variance (ANOVA) or two-way ANOVA followed by Tukey post hoc tests as appropriate. Mortality was analyzed by the Kaplan-Meier method. Significance was defined as *P* < 0.05.

## 3. Results

### 3.1. Pediatric DOX exposure-induced long-term cardiac dysfunction was attenuated by THZ1

To study the long-term effects of pediatric anthracycline exposure, 14-day-old mice received injections of DOX and were then monitored for another 32 weeks (Fig. 1A). Left ventricular ejection fraction (LVEF) was decreased as early as 1 week after the first DOX injection, and continued to decline gradually until the end of the study (Fig. 1B). DOX-induced declines in LVEF were attenuated by the CDK7/12/13 inhibitor THZ1, suggesting that THZ1 protected against DOX-induced systolic dysfunction. At 32 weeks after the first DOX injection, LV anterior wall thickness at end systole (LVAW;s) was significantly reduced (Fig. 1C). DOX-induced reduction in LVAW;s was attenuated by THZ1. LV posterior wall thickness at end diastole (LVPW;d) was significantly reduced by DOX, but was preserved when DOX was administered together with THZ1 (Table 1). Pediatric DOX treatment resulted in a significant increase in LV internal diameter at end systole (LVID;s), which was attenuated by THZ1 (Table 1). Pulsed wave Doppler revealed that DOX significantly reduced LV outflow tract (LVOT) peak velocity and velocity time integral (VTI), which were preserved by THZ1 (Fig. 1D, E). In addition, DOX induced a significant decrease in the early to late transmitral flow velocity ratio (E/A), indicating diastolic dysfunction (Fig. 1F). DOX-induced decrease in E/A ratio was completely prevented by THZ1. Taken together, pediatric DOX injection resulted in adulthood LV wall thinning, systolic and diastolic dysfunction, which appeared to be alleviated by THZ1.

### 3.2. Pediatric DOX-induced adulthood myocardial fibrosis was prevented by THZ1

**Fig. 2.**
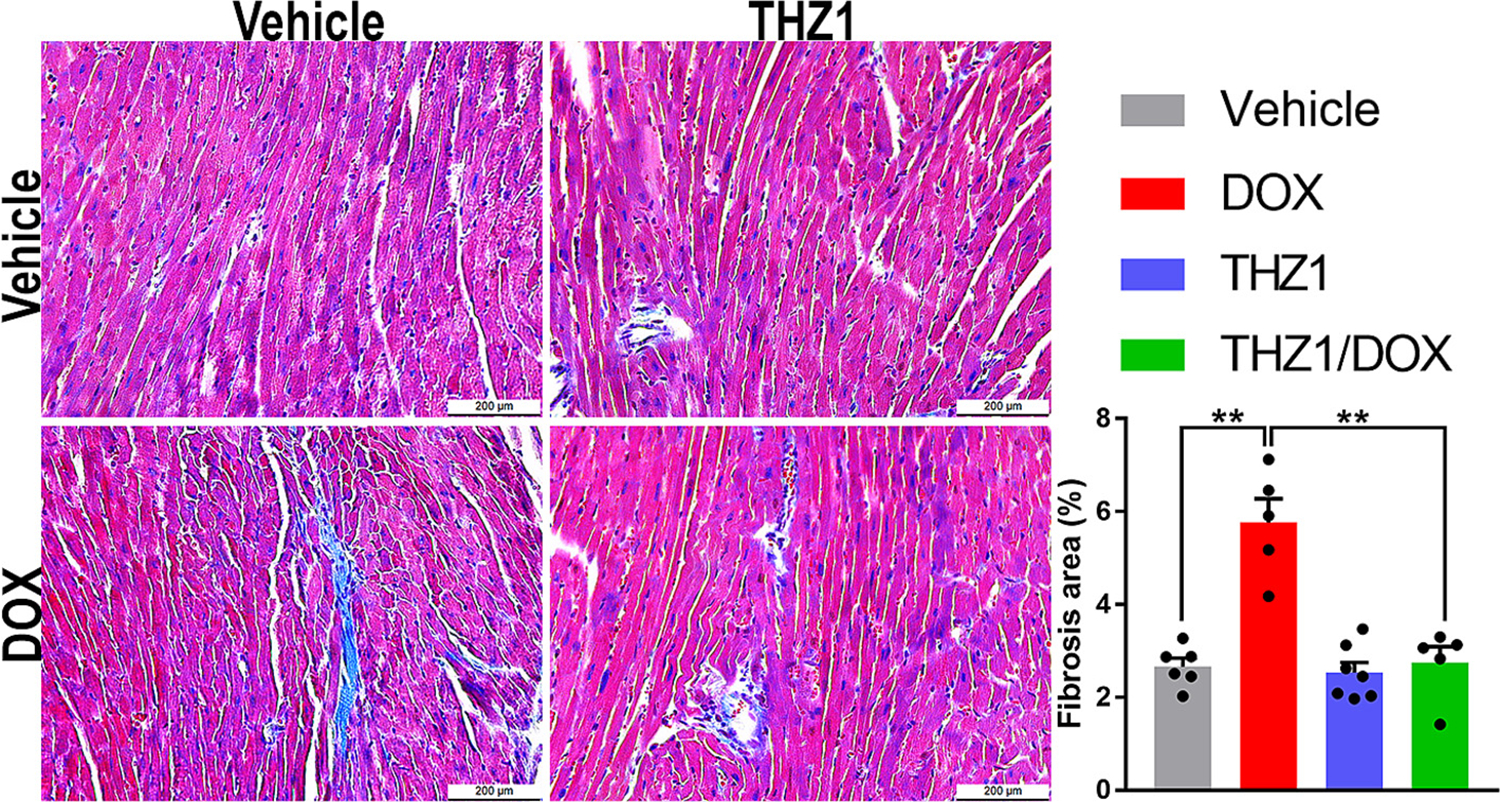
Pediatric DOX-induced myocardial fibrosis during adulthood was prevented by THZ1. Myocardial fibrosis (*blue*) at week 32 was evaluated by Masson’s trichrome staining. Scale bar = 200 μm. Vehicle (n = 6), DOX (n = 5), THZ1 (n = 7), THZ1/DOX (n = 5). Two-way ANOVA with Tukey’s test. ** *P* < 0.01.

Diastolic dysfunction can be caused by myocardial stiffness due to fibrosis. Indeed, pediatric DOX administration significantly increased interstitial fibrosis in the adult heart when compared with vehicle (Fig. 2). Pediatric DOX-related adulthood myocardial fibrosis was significantly reduced by THZ1. Myocardial fibrosis has been detected in long-term survivors of childhood leukemia, but the precise cause remains unknown (Cheung et al., 2015). Our findings suggest that pediatric exposure to DOX is sufficient to cause adulthood myocardial fibrosis, which can be prevented by treatment with THZ1.

### 3.3. THZ1 hindered pediatric DOX-induced decrease in cardiomyocyte size but not heart mass at adulthood

**Fig. 3.**
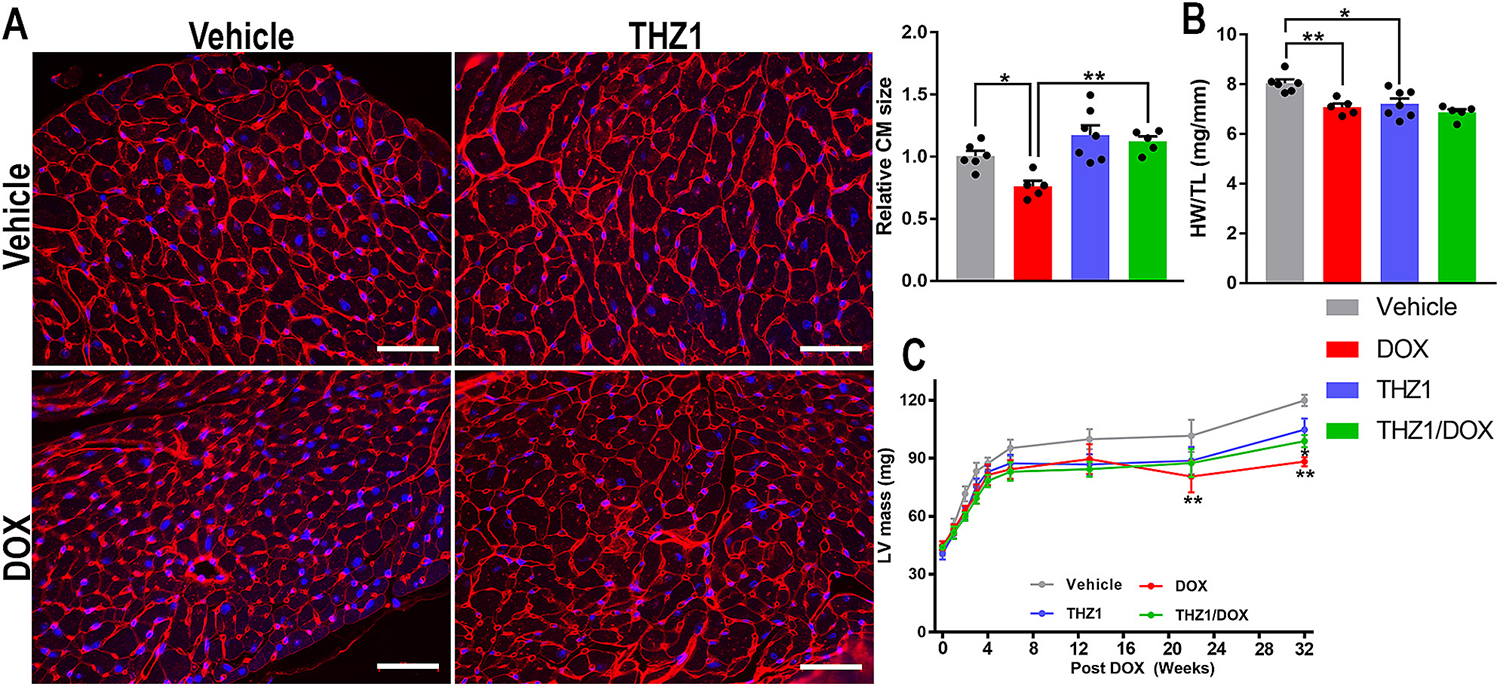
THZ1 hindered pediatric DOX-induced decrease in cardiomyocyte size but not heart mass at adulthood. (A) Cardiomyocyte cross-sectional area at week 32 was analyzed by staining with wheat germ agglutinin (WGA, *red*) and DAPI (*blue*). Scale bar = 50 μm. Vehicle (n = 6), DOX (n = 5), THZ1 (n = 7), THZ1/DOX (n = 5). (B) Heart weight/tibia length (HW/TL) at week 32. Vehicle (n = 6), DOX (n = 5), THZ1 (n = 7), THZ1/DOX (n = 5). Two-way ANOVA with Tukey’s test. * *P* < 0.05, ** *P* < 0.01. (C) LV mass. Vehicle (n = 6), DOX (n = 5–7), THZ1 (n = 7), THZ1/DOX (n = 5–7). Two-way ANOVA with Tukey’s test. * *P* < 0.05, ** *P* < 0.01 vs Vehicle.

We previously reported that DOX injection in adult mice induced cardiomyocyte atrophy (Xia et al., 2020). When compared with vehicle, administration of DOX in 14-day-old mice also reduced cardiomyocyte cross-sectional area 32 weeks later, indicating smaller cardiomyocytes (Fig. 3A). DOX-induced reduction in cardiomyocyte size was abolished by treatment with THZ1. Pediatric DOX administration also reduced heart weight/tibia length ratio, which was surprisingly not rescued by THZ1 (Fig. 3B). During pediatric and adolescent heart development within the first 6 weeks, echocardiography revealed a rapid increase in LV mass that was slightly delayed by pediatric DOX exposure, leading to a significant decrease in LV mass starting from week 22 (Fig. 3C). Pediatric DOX-induced decrease in LV mass was not attenuated by THZ1 (Fig. 3C). Collectively, THZ1 prevented pediatric DOX-induced decrease in cardiomyocyte size but not heart weight.

### 3.4. Pediatric THZ1 administration induced late cardiotoxicity

Despite the protection against DOX cardiotoxicity, THZ1 also displayed unexpected late adverse effects. Treatment with THZ1 alone at 14 days of age resulted in a significant decrease in LVEF starting from week 26 (Fig. 1B), suggesting that pediatric THZ1 exposure induced late-onset systolic dysfunction. Treatment with THZ1 in juvenile mice also significantly reduced heart weight/tibia length ratio at week 32 (Fig. 3B). In agreement with this result, pediatric THZ1 exposure delayed the increase in LV mass during development (Fig. 3C). Surprisingly, pediatric THZ1 induced a slight (but non-significant) increase in cardiomyocyte cross-sectional area at week 32 (Fig. 3A). Taken together, juvenile THZ1 treatment resulted in late cardiotoxicity that was likely associated with reduced cardiomyocyte number, via decreased proliferation or increased apoptosis.

### 3.5. Pediatric DOX-induced slow body weight gain and premature death were exacerbated by THZ1

**Fig. 4.**
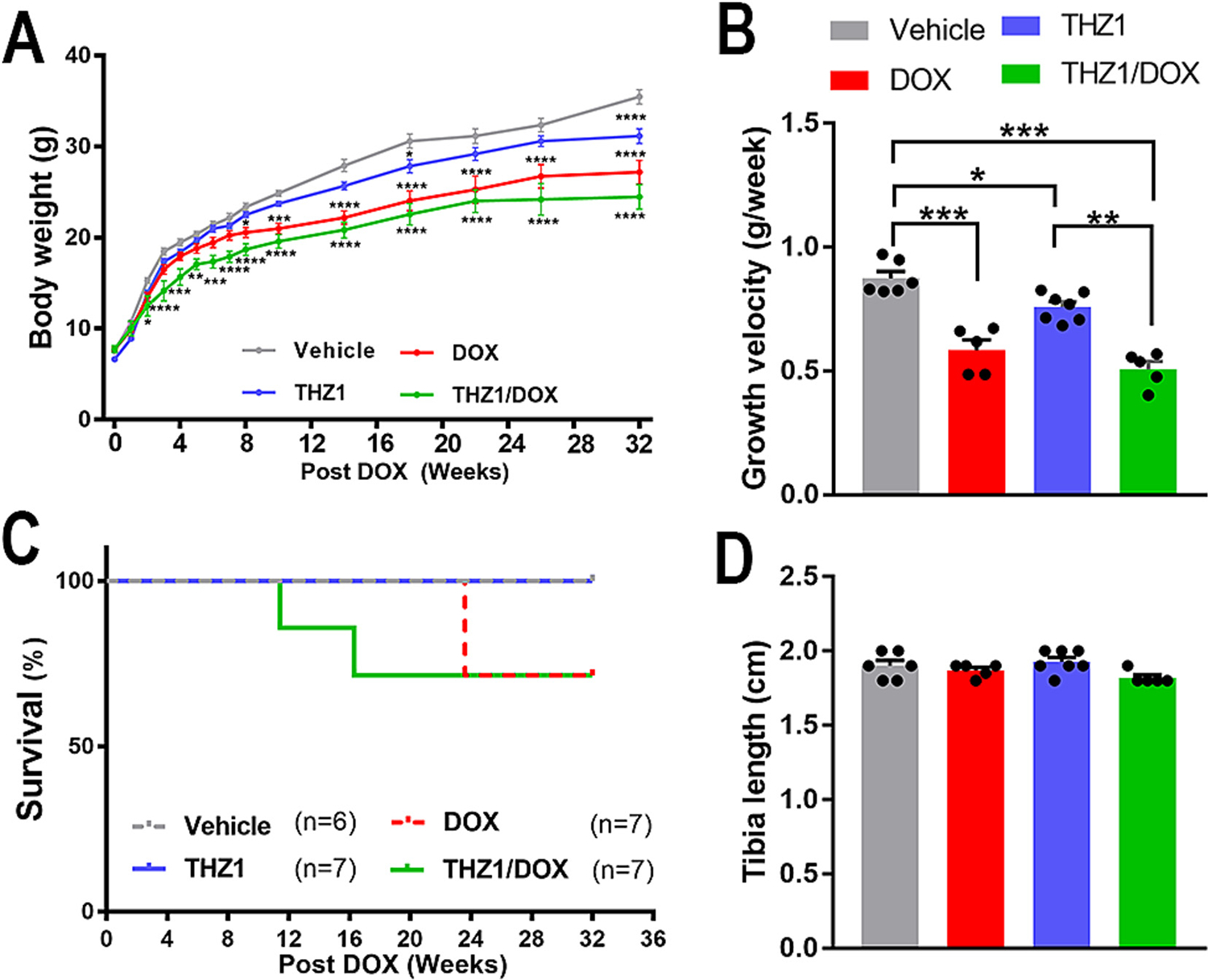
THZ1 failed to prevent pediatric DOX-induced delay in body weight gain. (A) Body weight. Vehicle (n = 6), DOX (n = 5–7), THZ1 (n = 7), THZ1/DOX (n = 5–7). Two-way ANOVA with Tukey’s test. * *P* < 0.05, ** *P* < 0.01, *** *P* < 0.001, **** *P* < 0.0001 vs Vehicle. (B) Body weight growth velocity during the study period. (C) Survival rate. (D) Tibia length at week 32. Vehicle (n = 6), DOX (n = 5), THZ1 (n = 7), THZ1/DOX (n = 5). Data were analyzed using Two-way ANOVA with Tukey’s test. * *P* < 0.05, ** *P* < 0.01, *** *P* < 0.001.

Adult survivors of pediatric malignancies are at a substantially higher risk of being underweight when compared with the general population (Meacham et al., 2005). Juvenile mice exposed to DOX also displayed a slower growth in body weight that can be detected at week 8 (Fig. 4A). Interestingly, combined treatment with DOX and THZ1 further exacerbated the delay in body weight growth, which reached statistical significance starting from week 2. Juvenile mice treated with THZ1 alone also exhibited a slower increase in body weight, but the difference did not reach statistical significance until week 32. The body weight growth velocity, defined as the body weight increase per week, was significantly reduced by treatment with THZ1, DOX or THZ1/DOX (Fig. 4B). The more substantial growth delay in the THZ1/DOX group was associated with accelerated mortality when compared to the DOX alone group (Fig. 4C). All mice in the vehicle and THZ1 group survived until the end of the study. Notably, tibia length was comparable among all groups at week 32 (Fig. 4D), suggesting that neither DOX nor THZ1 had a measurable effect on bone growth in length. Together, pediatric exposure to DOX or THZ1 delayed the growth in body weight but not bone length during physiological development.

### 3.6. THZ1 had negligible effects on pediatric DOX-induced growth delay in major organs

**Fig. 5.**
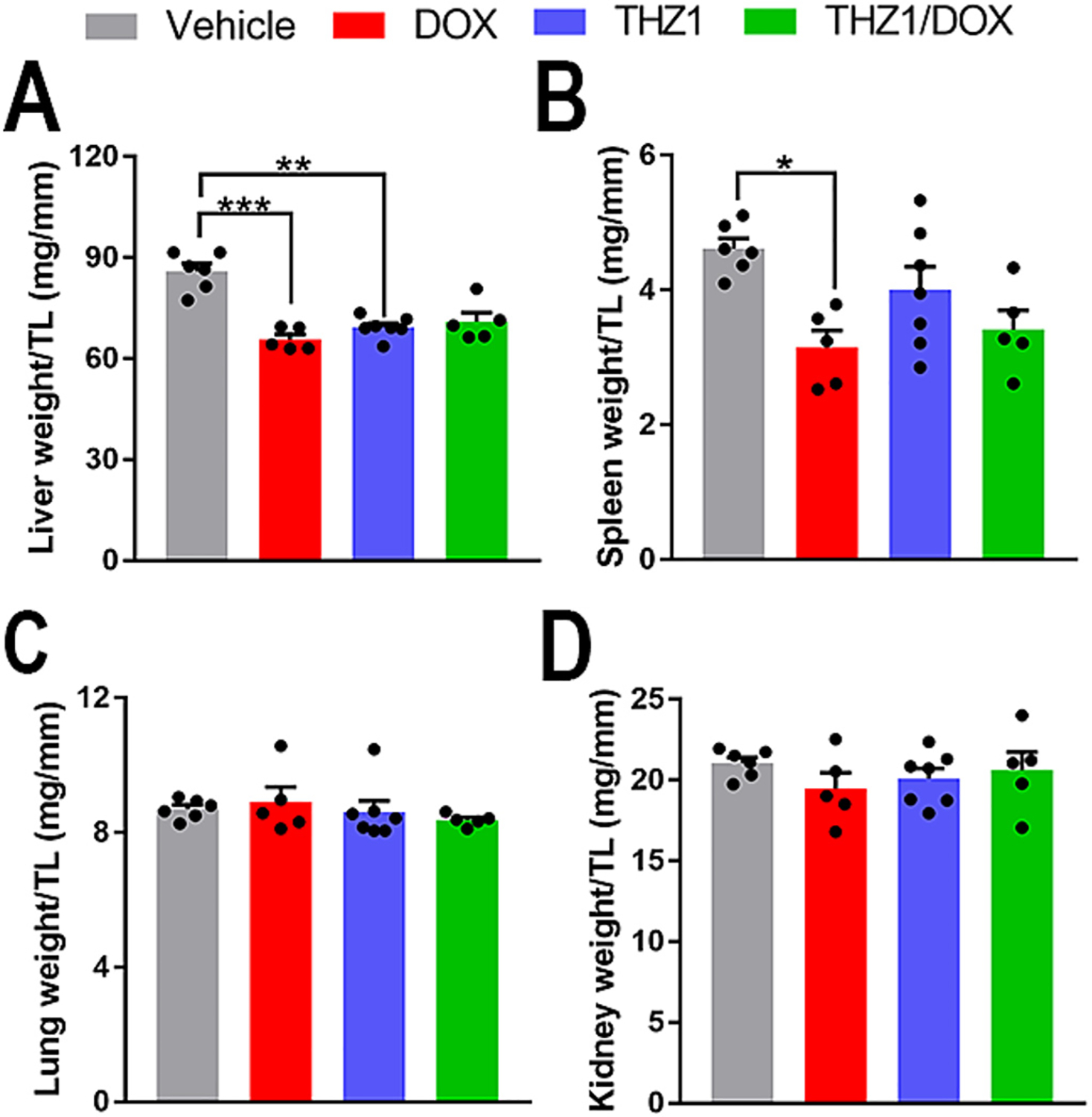
THZ1 had negligible effects on pediatric DOX-induced growth delay in major organs. The wet weight of major organs at week 32 was normalized to tibia length (TL). (A) Liver weight/TL ratio. (B) Spleen weight/TL ratio. (C) Lung weight/TL ratio. (D) Kidney weight/TL ratio. Vehicle (n = 6), DOX (n = 5), THZ1 (n = 7), THZ1/DOX (n = 5). Two-way ANOVA with Tukey’s test. * *P* < 0.05, ** *P* < 0.01, *** *P* < 0.001.

To further determine the effect of pediatric DOX exposure on the growth of major organs, the wet weight of liver, spleen, lung and kidney was measured at week 32. Compared with the vehicle group, pediatric DOX exposure significantly reduced adult liver weight (Fig. 5A) and spleen weight (Fig. 5B). Treatment with THZ1 failed to attenuate DOX-induced growth delay in liver and spleen. Interestingly, pediatric administration of THZ1 alone resulted in decreased liver weight during adulthood (Fig. 5A). The weight of lung or kidney during adulthood was not significantly altered by pediatric exposure to THZ1, DOX or the combination of THZ1 and DOX (Fig. 5C, D).

### 3.7. Pre-exposure to DOX impaired physiological cardiomyocyte hypertrophy via p38 activation

**Fig. 6.**
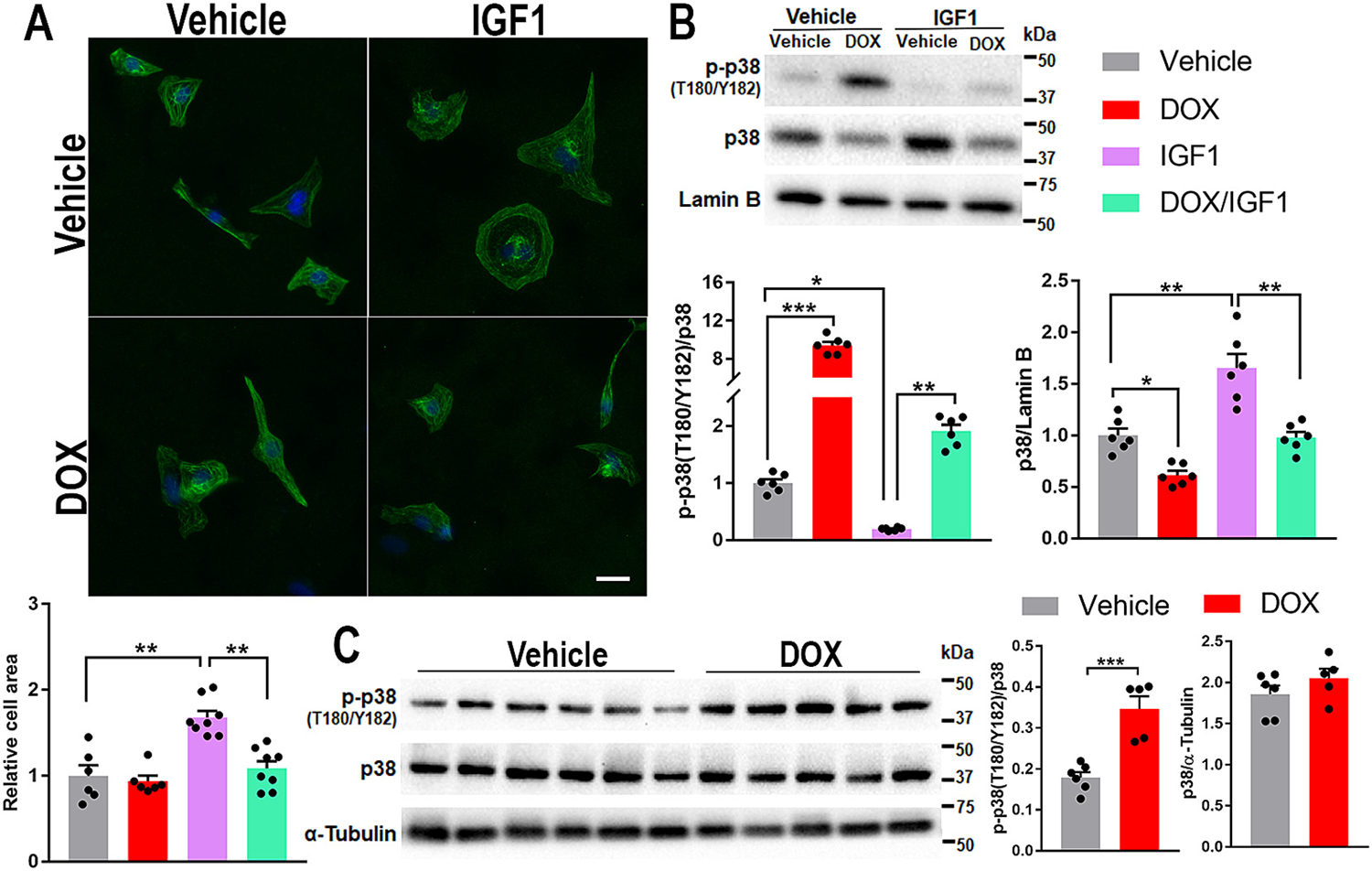
DOX induces prolonged p38 activation and suppresses physiological cardiomyocyte hypertrophy. (A,B) NRCMs were treated with DOX for 4 h, followed by insulin-like growth factor 1 (IGF1, 100 ng/ml) for 48 h. (A) Immunostaining for cardiac Troponin T (*green*) and nuclei (DAPI, *blue*). Scale bar = 10 μm. Cell surface area was measured using ImageJ. (B) Protein levels were analyzed by western blot. Two-way ANOVA with Tukey’s test. * *P* < 0.05, ** *P* < 0.01, *** *P* < 0.001. (C) Heart was harvested 32 weeks after pediatric DOX injection. Protein levels were analyzed by western blot. Vehicle (n = 6), DOX (n = 5). Student’s *t*-test. *** *P* < 0.001.

During postnatal development, the heart grows mainly through physiological cardiomyocyte hypertrophy, which is stimulated by growth factors such as insulin-like growth factor 1 (IGF1) (Nakamura and Sadoshima, 2018). To determine whether DOX pre-exposure diminishes physiological hypertrophy, neonatal rat cardiomyocytes (NRCMs) were pre-treated with DOX for 4 h, prior to stimulation with IGF1 for 48 h. IGF1-induced increase in cardiomyocyte size was significantly abrogated by DOX pre-exposure (Fig. 6A), suggesting that DOX pre-exposure hampered physiological cardiomyocyte hypertrophy. The p38 pathway has been shown to negatively regulate physiological hypertrophy (Taniike et al., 2008). Interestingly, pretreatment with DOX induced a significant, prolonged increase in phospho-p38 (Thr180/Tyr182), a marker of p38 activation (Fig. 6B). By contrast, IGF1-induced cardiomyocyte hypertrophy was associated with a marked downregulation of phospho-p38 (Thr180/Tyr182). IGF1-induced phospho-p38 (Thr180/Tyr182) downregulation was abolished by pretreatment with DOX (Fig. 6B), suggesting that pre-exposure to DOX may hamper physiological hypertrophy at least in part, through activation of p38. In agreement with this result, the level of phospho-p38 (Thr180/Tyr182) in mouse heart was significantly increased 32 weeks after pediatric DOX exposure (Fig. 6C).

## 4. Discussion

In the present study, we established a novel mouse model that may more closely mimic the long-term toxic effects of childhood DOX exposure. We demonstrated that pediatric DOX administration induced progressive cardiac dysfunction that was accompanied by structural changes including cardiac fibrosis and atrophy. Concurrent treatment with THZ1 alleviated the cardiotoxic effects of DOX. Surprisingly, THZ1 accelerated DOX-related mortality, which was associated with slower body weight gain during physical development and maturation. Among the previously reported mouse models of pediatric DOX cardiotoxicity (Zhu et al., 2008; Huang et al., 2010; Matsumura et al., 2018), our model is uniquely characterized by an extremely long follow up period of 32 weeks, which is, to our knowledge, the longest follow up of DOX toxicity in mice. This mouse model is valuable for understanding the chronic health challenges in adult survivors of childhood cancer.

Our pediatric mouse model for long-term DOX cardiotoxicity is clinically relevant in terms of age at exposure, dosing schedule, and follow-up period for late-stage cardiotoxicity.

1. Age at exposure: Mice from birth to 1 month of age develop >150 times faster than humans (Flurkey et al., 2007). In terms of the developmental stage, 14-day-old mice are roughly equivalent to 5-year-old children (14 mouse days X 150 ÷ 365 days/year = 5.8 human years). Therefore, an age of 14 days was selected for our pediatric mouse model.
2. Dosing schedule: The recommended DOX dosage for many human cancer treatments is 60–75 mg/m^2^ given intravenously every 21 days. Intravenous injection of a single dose of DOX (60 mg/m^2^) in humans results in similar pharmacokinetic profiles (*C*_max_, initial half-life, and terminal half-life) as a dose of DOX (5 mg/kg) in mice (van Asperen et al., 1999; Bramwell et al., 2002). To minimize the risk of cardiotoxicity, the cumulative dose of DOX in patients has generally been recommended to be limited below 250 mg/m^2^ (~4 injections at 60 mg/m^2^) when possible (Armenian et al., 2017). Therefore, previous studies in adult mice typically adopted a DOX injection protocol (5 mg/kg/week for 4 weeks, cumulative dose 20 mg/kg), which have been shown to cause cardiomyopathy within weeks regardless of the route of administration (i.v., or i.p.) (Li et al., 2016; Xia et al., 2023). Considering the technical challenges associated with repetitive intravenous injections in juvenile mice, intraperitoneal injection was used in this study. Due to slower drug clearance, young children are recommended to receive lower doses of DOX (Voller et al., 2017). In the Children’s Oncology Group Study AALL1131, children with newly diagnosed acute lymphoblastic leukemia were treated with a regimen including DOX (25 mg/m^2^) weekly for 3 weeks (Salzer et al., 2020), which is less than 50 % of the adult dosage of 60 mg/m^2^. Therefore, a dose of 2.5 mg/kg (50 % of the adult dose 5 mg/kg) was selected to induce pediatric DOX cardiotoxicity in mice. Cancer patients often receive DOX every 21 days to allow sufficient time for recovery from myelosuppression. A higher dosing frequency in mice (twice weekly for 2 weeks at 4 mg/kg, i.v.) has been reported to be adequate for recovery from DOX-induced decrease in blood cell count (Asmis et al., 2006). As 14-day-old mice mature rapidly, a dosing frequency of twice weekly for 2 weeks was selected to ensure all treatment is completed prior to adulthood.
3. Follow-up period for late-stage cardiotoxicity: Anthracycline exposure is associated with up to 5-fold higher incidence of congestive heart failure in childhood cancer survivors, even >20 years after cancer diagnosis (Mulrooney et al., 2009). Based on the standard lifespans in humans (~80 years) and mice (~2 years), 1 mouse year roughly equals 40 human years (Dutta and Sengupta, 2016). Therefore, a follow-up period of 32 weeks (~8 months) in mice is roughly equivalent to 27 years in humans (8 mouse months X 40 ÷ 12 months/year = 27 human years).

At present, the only cardioprotective drug approved by the U.S. Food and Drug Administration for anthracycline toxicity is dexrazoxane, which historically has not been widely used in pediatric cancer patients (de Baat et al., 2022). We recently demonstrated that THZ1 mitigated DOX-related cardiotoxicity and enhanced the anticancer efficacy of DOX in adult mice (Chen et al., 2024). Similar as THZ1, the CDK1/2/5 inhibitor roscovitine and the forkhead box O1 inhibitor AS1842856 also displayed protective effect against DOX cardiotoxicity in adult mice (Xia et al., 2018; Xia et al., 2020). A comparative study of dexrazoxane, THZ1, and other protective agents in our newly developed mouse model of pediatric DOX-induced late cardiotoxicity could provide critical insights into their long-term efficacy and safety.

Childhood cancer survivors received DOX decades ago display a progressive reduction in ventricular mass (Lipshultz et al., 2005; Armstrong et al., 2012), which was accurately recapitulated in our mouse model. Serial echocardiography revealed that pediatric DOX exposure hindered the increase in LV mass during normal growth and development. Inadequate ventricular mass, characterized by reduced wall thickness and chamber dimension, has been suggested as the primary cause of excess afterload that eventually leads to cardiomyopathy in adult survivors of childhood cancer (Lipshultz et al., 2005). Therefore, understanding the molecular and cellular mechanisms of myocardial mass reduction is crucial. We showed that pediatric DOX-induced ventricular mass reduction was associated with a decrease in cardiomyocyte size at week 32. As postnatal heart grows primarily through physiological cardiomyocyte hypertrophy, our findings suggested that childhood DOX exposure may interfere with physiological hypertrophy during development at least in part, through prolonged activation of p38 in cardiomyocytes.

Despite the partial protection by THZ1 against DOX-induced late cardiotoxicity, the combination of THZ1 with DOX resulted in accelerated mortality in mice. A potential explanation is that combined treatment with THZ1 and DOX resulted in slower body weight gain during pediatric and adolescent development. Indeed, a most recent report found that underweight was associated with a higher risk of late mortality in childhood cancer survivors (Tonorezos et al., 2024b). Our mouse model may be employed in future studies to test whether pediatric DOX-related mortality can be attenuated by healthy weight gain through increased caloric intake.

Underweight is more prevalent in childhood cancer survivors than in age- and sex-matched controls (Meacham et al., 2005; Brouwer et al., 2012). Particularly, childhood cancer survivors treated with anthracyclines have a slower increase in body mass index (BMI) after completion of treatment, resulting in an increased prevalence of underweight (Meacham et al., 2005; Brouwer et al., 2012). In agreement with these clinical findings, the current study also revealed that pediatric DOX exposure reduced growth velocity and body weight in mice. It is noteworthy that certain cancer therapies (e.g. cranial radiation therapy) are associated with a greater increase in BMI (i.e. overweight, or obesity) in childhood cancer survivors (Meacham et al., 2005; Garmey et al., 2008; Brouwer et al., 2012).

Childhood cancer survivors have decreased heights during adulthood (Chow et al., 2007). Major risk factors for adult short stature include young age at diagnosis and craniospinal radiation involving the hypothalamic-pituitary axis (Gurney et al., 2003; Chow et al., 2007; Knijnenburg et al., 2013). Indeed, hypothalamic-pituitary disorders are quite common in childhood cancer survivors and are known to cause adverse outcomes including short stature (van Iersel et al., 2019). Although chemotherapy contributes to decreased adult height, a dose-effect relationship between any chemotherapeutic agent and short stature cannot be detected (Chow et al., 2007). In another study, chemotherapy was not associated with the risk for adult short stature (Gurney et al., 2003). Our study provides the first, direct evidence that pediatric DOX exposure does not interfere with growth in bone length in mice, suggesting that anthracyclines are likely not a major cause of the decreased heights in adult survivors of childhood cancer.

While DOX is well-documented for its cardiotoxic effect, its impact on other major organs remains poorly understood, especially after a prolonged latent period following pediatric exposure. In this study, we demonstrated that pediatric DOX exposure reduced the weights of liver and spleen, but not those of lung and kidney at week 32. In agreement with our findings, adulthood DOX exposure has been shown to induce hepatocyte vacuolation and focal necrosis in the liver (Prasanna et al., 2020). Moreover, treatment with DOX in adult mice also induced splenic atrophy (Jadapalli et al., 2018; Xue et al., 2024). Collectively, these findings suggest that pediatric DOX exposure may have late toxic effects on the liver and spleen, which may warrant careful monitoring and further investigation in the future.

Our study has several limitations. To obtain meaningful data with the minimum number of animals, only male mice (*n* = 5–7 per group) were used in this study. While the sample size appeared sufficient to detect statistically significant differences in most experiments, it is possible that additional differences might have reached statistical significance had more mice been included. To prioritize the assessment of long-term toxicity and simplify the study design, sex as a biological variable was not investigated. At present, clinical studies have yielded mixed results regarding the effect of sex on anthracycline cardiotoxicity (Wilcox et al., 2022). It might be interesting to explore potential sex-based differences in our mouse model of pediatric DOX-induced late cardiotoxicity in the future. In addition, only one dosing schedule of THZ1 was evaluated. In our previous study with adult mice, THZ1 was administered at a dose of 10 mg/kg (twice daily, 5 days/week for 5 weeks) (Chen et al., 2024). For the juvenile mice in this study, THZ1 was administered at a dose of 10 mg/kg (twice daily, 4 days per week for 2 weeks), which represents a lower frequency and shorter treatment period. Despite the partial protection against DOX cardiotoxicity, pediatric THZ1 alone reduced heart, liver and body weight with a trend of increased cardiomyocyte size at week 32, indicating late toxic effects. Therefore, the dosing schedule of THZ1 may need to be optimized to maintain cardioprotection but reduce its adverse effects in future studies. Initially developed as a covalent CDK7 inhibitor, THZ1 may exhibit cardioprotection and toxicity through on-target inhibition of CDK7 and/or off-target inhibition of CDK12/13 (Kwiatkowski et al., 2014). Compared with THZ1, non-covalent selective CDK7 inhibitors (e. g. samuraciclib, SY-5609, currently in clinical development) may have better safety profiles. As CDK7 activation contributes to the pathogenesis of various diseases (Belew et al., 2025), the effect and safety of selective CDK7 inhibition on pediatric DOX-related late cardiotoxicity warrants further investigation in future studies.

In conclusion, we showed that pediatric DOX exposure induces long-term pathological changes in various organs including the heart, liver and spleen when measured at adulthood. Treatment with THZ1 partially prevented DOX-induced cardiotoxicity, but did not necessarily attenuate other late effects of DOX. Our mouse model of pediatric DOX-associated late toxicity closely mimics the clinical observations in adult survivors of childhood cancer. This new mouse model is extremely useful for studying the late effects of childhood DOX exposure. In addition, our mouse model may also facilitate the development of novel strategies to protect children with cancer from developing cancer treatment-related long-term toxicity.

## Supplementary Material

1

## Figures and Tables

**Table 1:** Echocardiography 32 weeks after pediatric DOX injection.

Group	Vehicle (*n* = 6)	DOX (*n* = 5)	THZ1 (*n* = 7)	THZ1/DOX (n = 5)
LVAW;d (mm)	0.63 ± 0.02	0.56 ± 0.03	0.62 ± 0.02	0.61 ± 0.02
LVAW;s (mm)	1.00 ± 0.06	0.72 ± 0.03**	0.93 ± 0.03	0.88 ± 0.07
LVID;d (mm)	4.44 ± 0.04	4.20 ± 0.16	4.24 ± 0.11	4.19 ± 0.12
LVID;s (mm)	2.99 ± 0.04	3.50 ± 0.15*	3.10 ± 0.09	3.22 ± 0.11
LVPW;d (mm)	0.79 ± 0.03	0.65 ± 0.03*	0.73 ± 0.03	0.71 ± 0.04
LVPW;s (mm)	1.03 ± 0.08	0.87 ± 0.04	1.02 ± 0.03	0.97 ± 0.07
LVEF (%)	61.22 ± 0.85	35.18 ± 1.38****	52.6 ± 1.5***	46.78 ± 1.2^####^
LVFS (%)	32.66 ± 0.61	16.63 ± 0.71****	26.79 ± 0.96***	23.16 ± 0.68^###^
LV Vol;d (μl)	89.54 ± 1.98	79.27 ± 7.12	80.86 ± 5	78.44 ± 5.31
LV Vol;s (μl)	34.72 ± 1.05	51.56 ± 5.16**	38.39 ± 2.85	41.89 ± 3.43

Abbreviations: LVAW;d, LV end-diastolic anterior wall thickness; LVAW;s, LV end-systolic anterior wall thickness; LVID;d, LV end-diastolic internal diameter; LVID;s, LV end-systolic internal diameter; LVPW;d, LV end-diastolic posterior wall thickness; LVPW;s, LV end-systolic posterior wall thickness; LVEF, LV ejection fraction; LVFS, LV fractional shortening; LV Vol;d, LV end-diastolic volume; LV Vol;s, LV end-systolic volume. All data were analyzed using two-way ANOVA with Tukey’s test.

* *P* < 0.05,

** *P* < 0.01,

*** *P* < 0.001,

**** *P* < 0.0001 vs. Vehicle;

### *P* < 0.001,

#### *P* < 0.0001 vs. DOX.

## Data Availability

No data was used for the research described in the article.

## Appendix A. Supplementary data

Supplementary data to this article can be found online at https://doi.org/10.1016/j.taap.2025.117600.

## Abbreviations:

CDK7: cyclin-dependent kinase 7
DOX: doxorubicin
EF: ejection fraction
FS: fractional shortening
NRCMs: neonatal rat cardiomyocytes
