# Supplementary figures and images for: Pediatric doxorubicin exposure induces persistent pathological changes in mice
Source: Toxicol Appl Pharmacol. Author manuscript; Available in PMC 2026 Jun 15. (PMC13264827; doi:10.1016/j.taap.2025.117600)

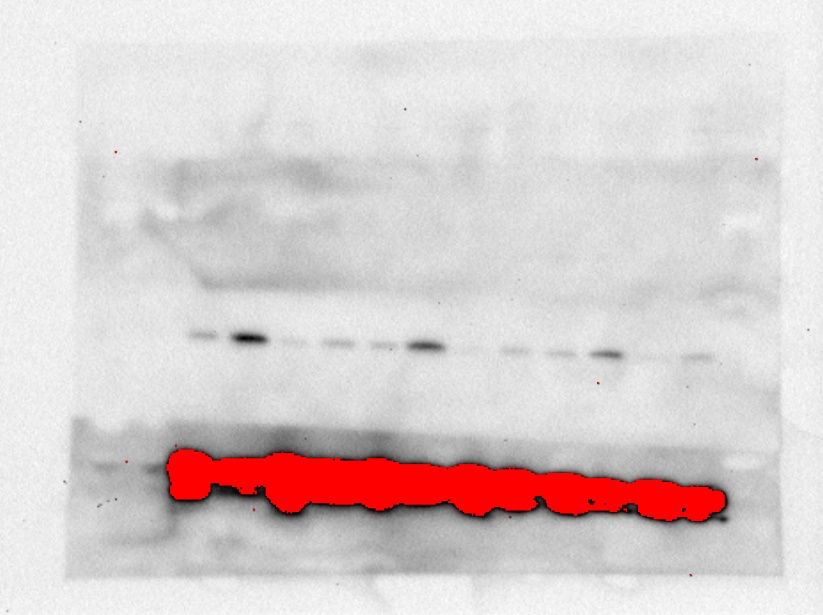
**Figure 6B**


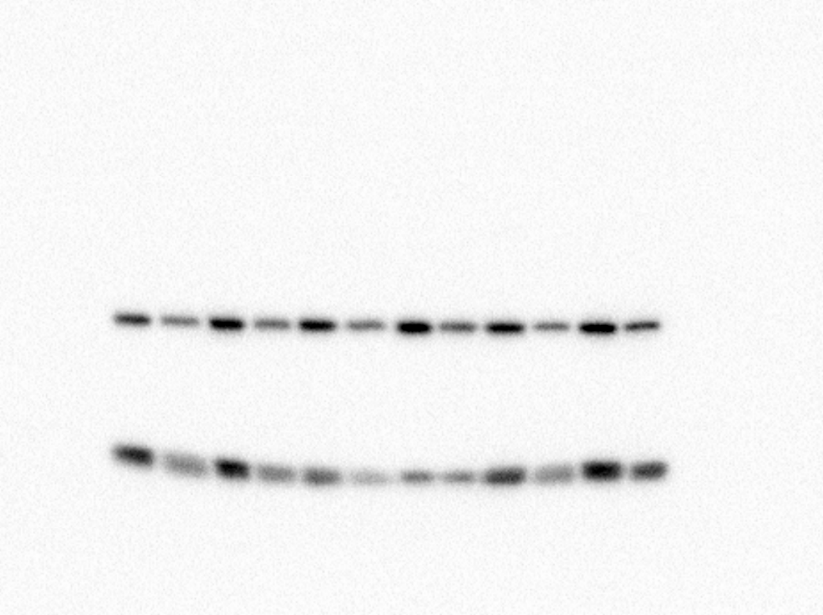


**p38**

**38kDa**

**p-p38** **(T180/Y182)**

**38kDa**


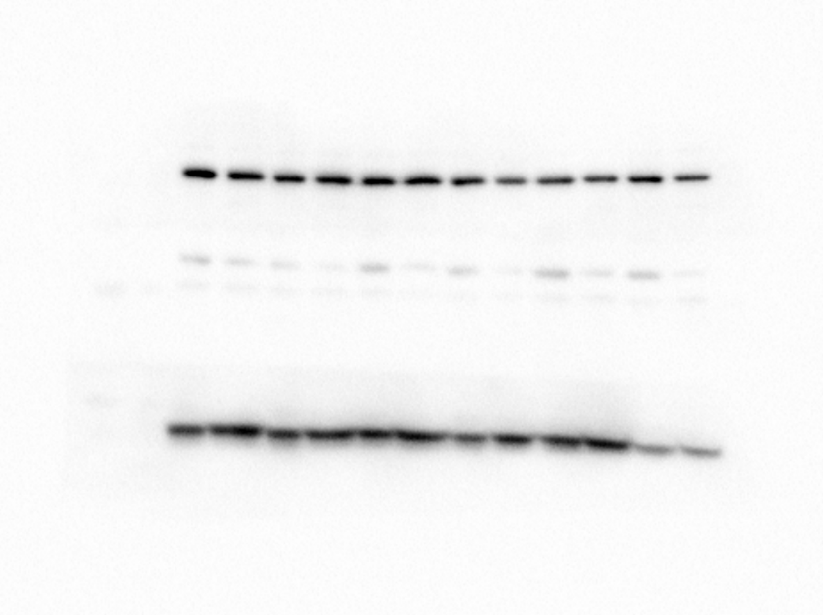


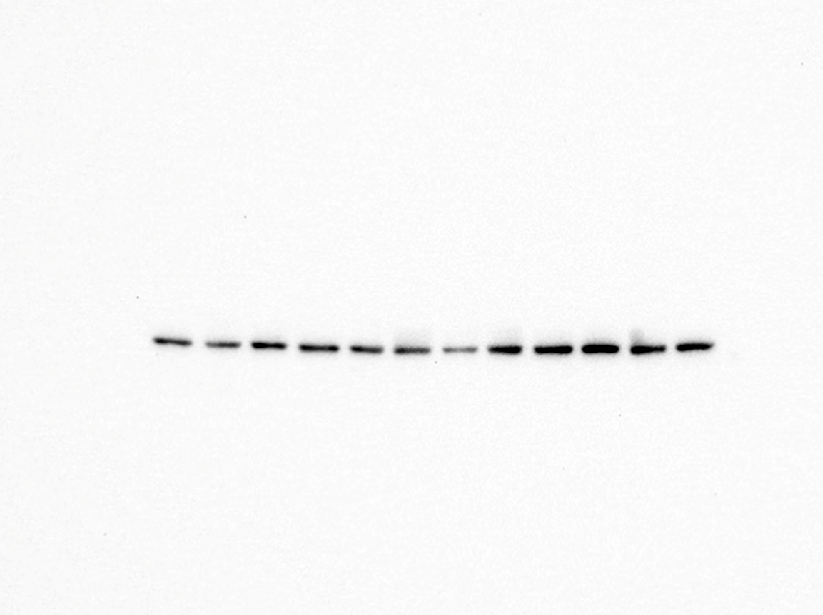


**Figure 6C**

**p-p38** **(T180/Y182)**

**38kDa**

**Lamin B**

**68kDa**


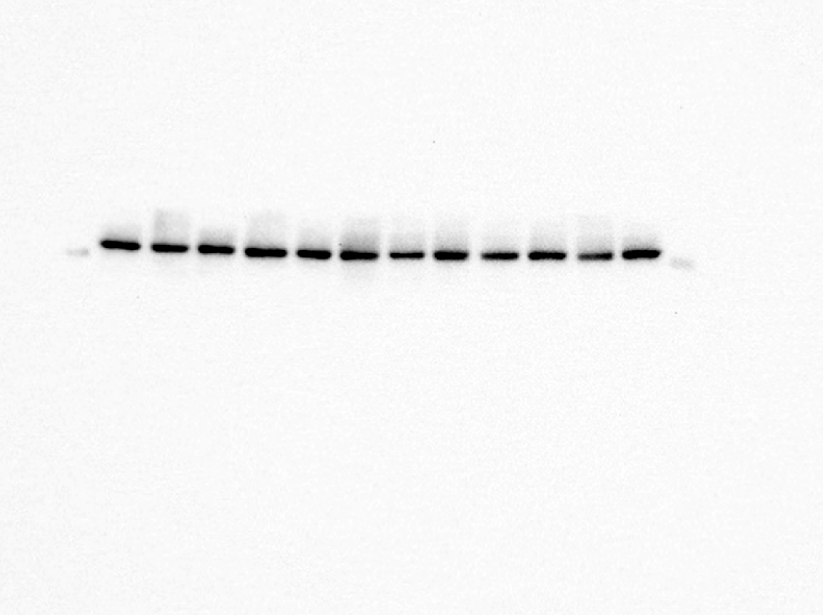


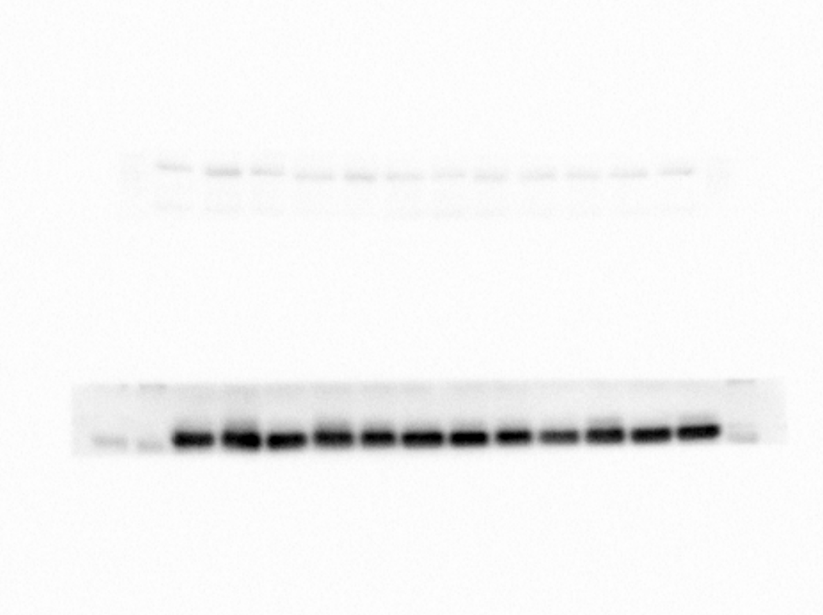


**α-Tubulin**

**50kDa**

**p38**

**38kDa**

Supplement: 1 [file NIHMS2184841-supplement-1.docx]
